# Supplementary material for: Efficient Generation of Functional TCRαβ+ Cytotoxic T Cells from hiPSCs via Small-Molecule Modulation
Source: bioRxiv. 2026 Apr 1:2026.03.31.715684. Preprint. [Version 1] doi: 10.64898/2026.03.31.715684 (PMC13060064; doi:10.64898/2026.03.31.715684)
Supplement: 1 [file NIHPP2026.03.31.715684v1-supplement-1.pdf]

bioRxiv preprint doi: https://doi.org/10.64898/2026.03.31.715684; this version posted April 1, 2026. The copyright holder for this preprint (which was not certified by peer review) is the author/funder, who has granted bioRxiv a license to display the preprint in perpetuity. It is made available under aCC-BY 4.0 International license.

subtracting 10% of the C02 signal from the C05 signal. Measurement results were analyzed using Python in Google Colab. For screening validation experiments drugs were either added once at the iT day 14 ProT cell stage at the following concentrations (GNF351 1 μM, CH223191 1 μM, EPZ004777 1 μM, OAC1 5 μM, CHIR99021 3 μM) or replenished with every half media change (every 3-4 days).

Flow cytometry

For *in vitro* differentiation experiments, 50-100 μl of sample were collected from wells and filtered through a filter-top polystyrene flow cytometry tube, pelleted at 300g for 5min and resuspended in flow cytometry buffer (PBS+ 2%FBS) containing the desired antibody cocktail (antibodies are listed in table S2). After 20 min incubation at room temperature in the dark, cells were washed with flow buffer and resuspended in flow buffer containing viability dye (PI, DAPI or Sytox Blue). Sample acquisition was performed on a SONY MA900, LSRFortessa (BD) or Cytek Aurora and analyzed using FlowJo v10.10.0. For zebrafish experiments, pools of zebrafish embryos/larvae were dechorionated and resuspended in 500 ml Liberase TM (Millipore Sigma) solution (75 mg/ml in 1xPBS/1mM EDTA), incubated at 34C and dissociated with a 1000 ml pipette every 15-30 minutes until embryo trunks are no longer visible (Frame et al., 2020); each data point represents 6 zebrafish. $CD41^{+}$ zebrafish HSPCs were gated separately from the CD41hi thrombocyte population (Lin et al., 2005; Ma et al., 2011).

Table S2

**Target**	**Fluorochrome**	**Vendor**	**Cat. No.**	**Clone**
CD3	PE-Cy7	Biolegend	344816	SK7
CD4	PE-Cy5	BD	555348	RPA-T4
CD5	BV510	BD	563381	UCHT2
CD7	PE	BD	555361	M-T701
CD8	BV421	BD	562428	RPA-T8
CD45	APC-Cy7	BD<br>Biolegend	557833<br>304014	2D1<br>HI30
TCRα/β	APC	Biolegend	306717	IP26
CD56	FITC	Biolegend	362546	5.1H11
CD19	PE	Biolegend	302207	HIB19

B cell differentiation

bioRxiv preprint doi: https://doi.org/10.64898/2026.03.31.715684; this version posted April 1, 2026. The copyright holder for this preprint (which was not certified by peer review) is the author/funder, who has granted bioRxiv a license to display the preprint in perpetuity. It is made available under aCC-BY 4.0 International license.

Full 8x sequence insert sequence:

aaaaaggtaccACCTGCAGATCTCTAGAAGCTTGCAGTAGCGTGCGATGTCTCAAGCTTGCAGTAGCGTGCGATGTCTCAAGCTTGCAGTAGCGTGCGATGTCTCAAGCTTGCAGTAGC.GTGCGATGTCTCAAGCTTGCAGTAGCGTGCGATGTCTCAAGCTTGCAGTAGCGTGCGATGTCTCAAGCTTGCAGTAGCGTGCGATGTCTCAAGCTTGCAGTAGCGTGCGActgcaagcttctagagatctgcaggtcgaggtcgacggtatcgatGGATCCAGAGGGTATATAATGGAAGCTCGACTTCCAGCTCGAccgcggaaaaa

Table S3

**Recombinant DNA**	**Source**	**Identifier**
Plasmid: p5E-8XRE	This paper	
Plasmid: pME-GFP	Kwan et al., 2007	Tol2kit v1.2 #237
Plasmid: p3E-polyA	Kwan et al., 2007	Tol2kit v1.2 #302
Plasmid: pDestTol2pA2	Kwan et al., 2007	Tol2kit v1.2 #394

Zebrafish Embryo Exposure

Groups of 6 stage-matched embryos were arranged in 12 well plates in 2mL E3 water with or without chemical treatments of interest. Zebrafish were dosed with DMSO (1um), FICZ (10nm), CH223191 (1um) and/or OAC1 (1um).

Statistics

Statistic tests were performed using GraphPad Prism Version 11.0.0. Statistical significance is indicated as *: $P \leq 0.05$, **: $P \leq 0.01$, ***: $P \leq 0.001$, ****: $P \leq 0.0001$. For comparison of two groups unpaired t-tests with Welch's correction were used, in cases where more than 2 groups were compared Ordinary one-way ANOVA was performed.

ACKNOWLEDGMENTS

This work was supported by NIH fund NIDDK R01DK134515 (RGR), 1R01HL154580 (TEN), and 1RC2DK120535-01A1 (TEN, GQD). Funding for the Cell Voyager imager was provided by

bioRxiv preprint doi: https://doi.org/10.64898/2026.03.31.715684; this version posted April 1, 2026. The copyright holder for this preprint (which was not certified by peer review) is the author/funder, who has granted bioRxiv a license to display the preprint in perpetuity. It is made available under aCC-BY 4.0 International license.

SUPPLEMENTAL FIGURE 1

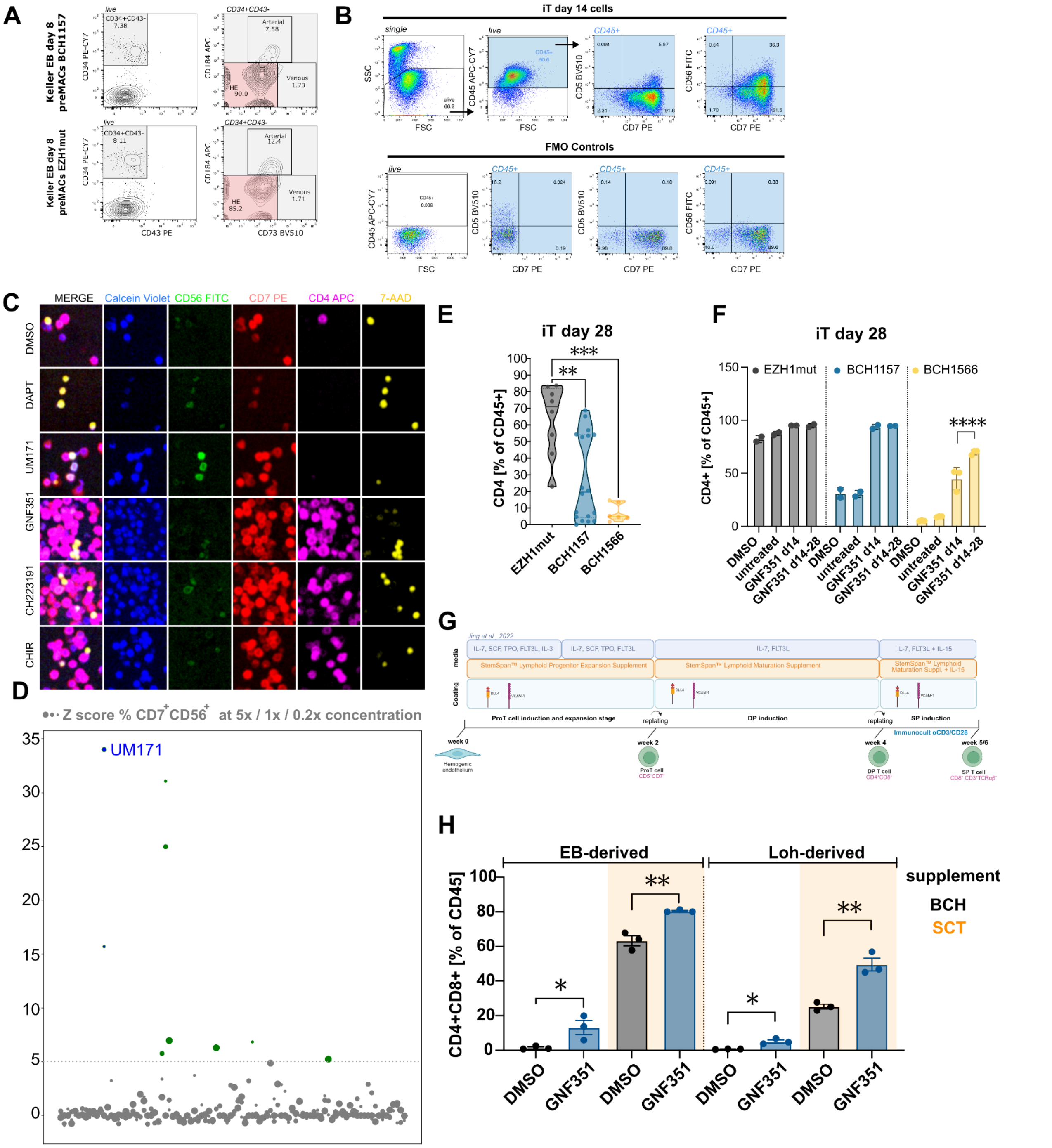

bioRxiv preprint doi: https://doi.org/10.64898/2026.03.31.715684; this version posted April 1, 2026. The copyright holder for this preprint (which was not certified by peer review) is the author/funder, who has granted bioRxiv a license to display the preprint in perpetuity. It is made available under aCC-BY 4.0 International license.

FIG S1

A: Representative flow cytometric profiling of hiPSC-derived hemogenic endothelial cells (CD34+CD43-CD184-CD73-) from BCH1157 and EZH1mut hiPCSs prior to CD34+ MACS enrichment. B: Exemplary gating strategy for flow cytometric analysis of iT day 14 cells. C: Fluorescent-microscopic image of iT day 21 cells after antibody staining against CD56 (FITC), CD7 (PE), CD4 (APC) including viability dyes Calcein Violet and 7-AAD. Scale bar indicates 20 μM.

D: Z scores for CD56-inducing hits (potential NK cell inducing agents). Compounds with Z-scores higher than 5 were considered hits. E: Percentage of CD4+ cells on iT day 28 in 3 hiPSC lines. Each data point represents one well of iT cell differentiation. F: $CD4^+$ percentage in iT differentiation day 28 cultures from 3 hiPSC treated with GNF351 either as a single dose on iT day 14 or continuously from iT day 14 to 28. Bar graphs depict mean with SEM. G: Schematic of iT differentiation protocol timeline using commercial media vs media published in Jing et al. 2022,

H:GNF351 has a positive effect on $CD4^+CD8^+$ independent of HSPC protocol and iT differentiation medium. Percentage of $CD4^+CD8^+$ cells on iT day 28 in hiPSC line BCH1157 using different protocols for the derivation of hematopoietic progenitors and different iT differentiation culture conditions.

bioRxiv preprint doi: https://doi.org/10.64898/2026.03.31.715684; this version posted April 1, 2026. The copyright holder for this preprint (which was not certified by peer review) is the author/funder, who has granted bioRxiv a license to display the preprint in perpetuity. It is made available under aCC-BY 4.0 International license.

SUPPLEMENTAL FIGURE 2

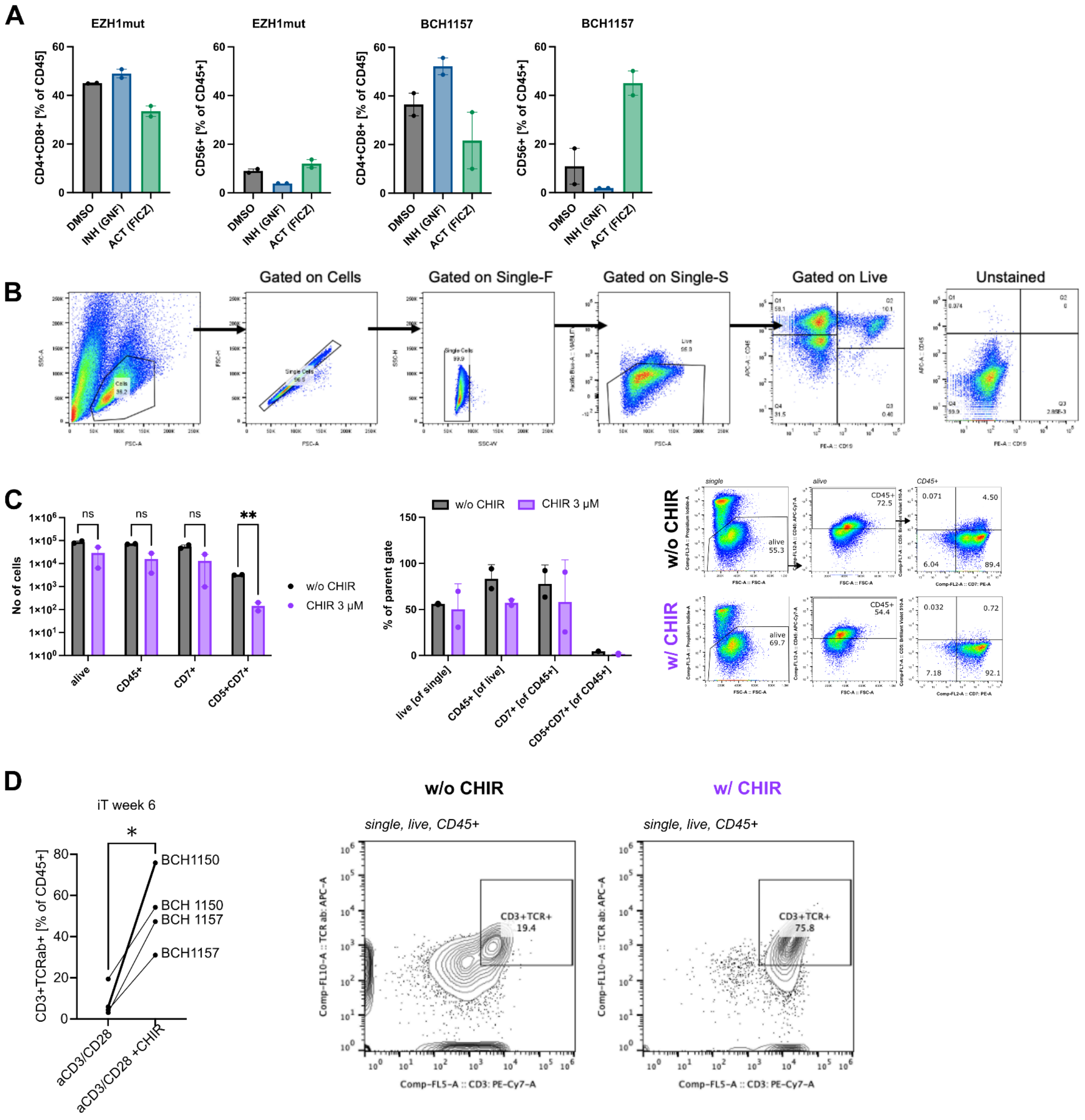

FIG S2 Importance of timing in AHR and WNT modulation

A: Flow cytometric quantification of $CD4^{+}CD8^{+}$ or $CD56^{+}$ expression at 4 weeks of iT differentiation in the presence of AHR inhibitor (GNF, 1 μM) or AHR activator (FICZ, 0.2 μM) in two hiPSC lines (EZH1mut or BCH1157).

B: Gating strategy for flow cytometric assessment of CB $CD34^{+}$ differentiation outcome towards B cells.

C: Flow cytometric profiling of hiPSC to ProT cell differentiation with and without CHIR addition. C: Percentage of $CD3^{+}TCRab^{+}$ cells after single positive induction with anti CD3/CD28 antibody with and without CHIR addition.

bioRxiv preprint doi: https://doi.org/10.64898/2026.03.31.715684; this version posted April 1, 2026. The copyright holder for this preprint (which was not certified by peer review) is the author/funder, who has granted bioRxiv a license to display the preprint in perpetuity. It is made available under aCC-BY 4.0 International license.

FIGURE S3

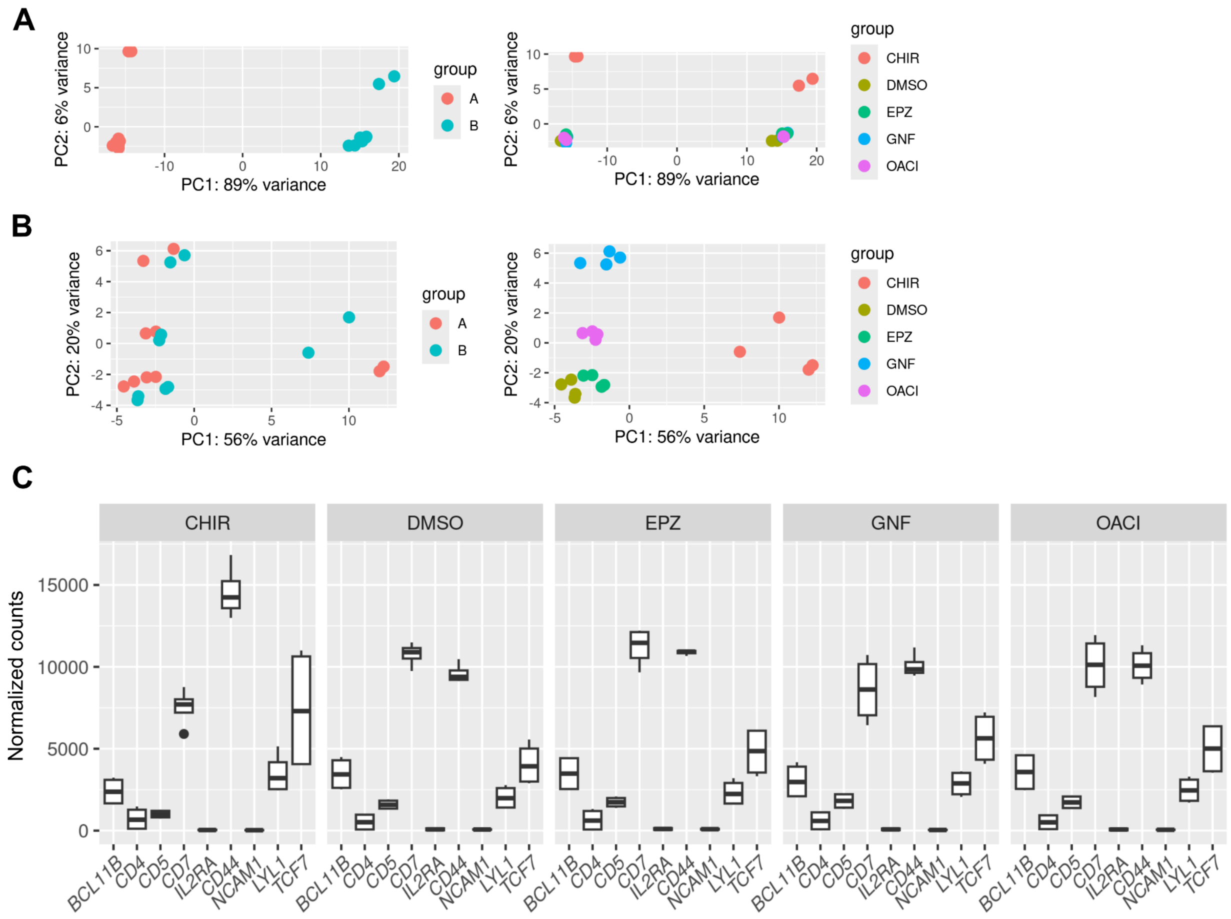

FIG S3: Related to Figure 3

A: Principal component analysis (PCA) of drug treated ProT cells derived from cord blood donor A and B before batch correction and after (B). C: Expression level of ProT cell genes 24h post small molecule treatment

bioRxiv preprint doi: https://doi.org/10.64898/2026.03.31.715684; this version posted April 1, 2026. The copyright holder for this preprint (which was not certified by peer review) is the author/funder, who has granted bioRxiv a license to display the preprint in perpetuity. It is made available under aCC-BY 4.0 International license.

FIGURE S4

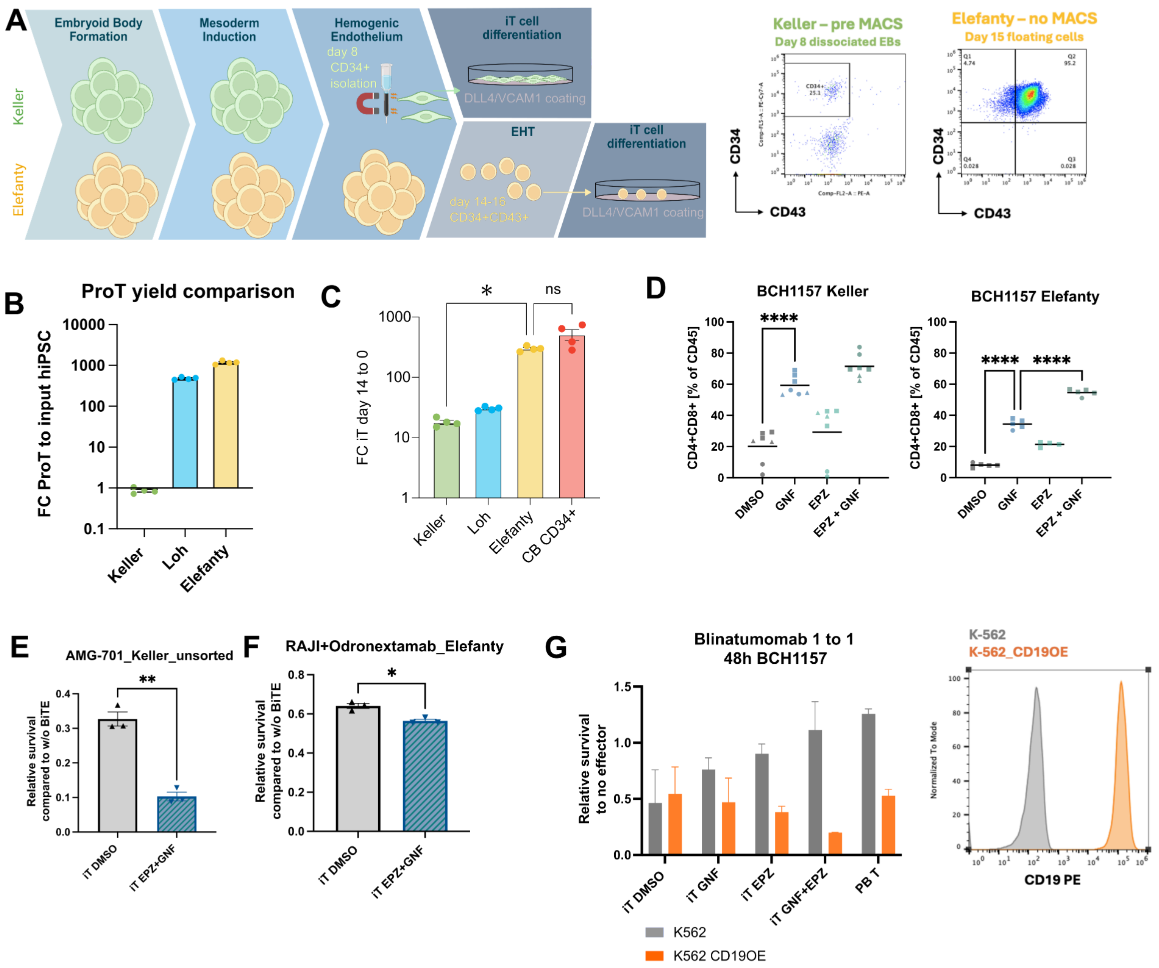

FIG S4: Related to Figure 4

A: Schematic timeline of Keller compared to Elefanty EB differentiation and representative flow plots of input cells for IT differentiation. B: Quantification of fold increase of ProT cells versus input hiPSCs for Keller, Loh and Elefanty hematopoietic differentiation protocols. C: Quantification of fold expansion of plated day 0 cells compared to cell counts on iT day 14 for indicated differentiation protocols and CB CD34^{+} cells. D: Additional experimental data for FIG 4B showing percentage of CD4^{+}CD8^{+} double positive cells differentiated from hiPSC line BCH1157 using either the Keller or Elefanty protocol. Each data point represents one well, data from the same differentiation round are indicated by the same shape. One-way ANOVA. E: Relative survival of target cells to no BiTE ctrls after 48h of co-culture with iT cells derived in the presence of small molecule inhibitors without prior enrichment for CD8 SP cells. AMG-701 was added at 3nM F: Relative survival of target cells (Raji) to no BiTE ctrls after 48h of co-culture with iT cells derived in the presence of small molecule inhibitors without prior enrichment for CD8 SP cells. Odronextamab was added at 1nM. G: Relative survival to no effector ctrl of iT cells derived with and without small molecule treatment after 48h of co-culture with K-562 (grey) or K-562 CD19 overexpressing cells (orange) in the presence of 1ng/ml Blinatumomab. Data are from triplicate wells. Flow plot shows CD19 staining of K-562 and K-562 CD19OE cells.

bioRxiv preprint doi: https://doi.org/10.64898/2026.03.31.715684; this version posted April 1, 2026. The copyright holder for this preprint (which was not certified by peer review) is the author/funder, who has granted bioRxiv a license to display the preprint in perpetuity. It is made available under aCC-BY 4.0 International license.

Supplemental Table S1

agent	stock solvent	vendor	stock concentration [μM]
16,16dmPGE2	DMSO	tocris	10000
53AH	DMSO	cellagen	6000
740 Y-P	DMSO	medchemexpress	5000
A-769662	DMSO	tocris	50000
AF12198	DMSO	tocris	5000
Afatinib	DMSO	selleckchem	5000
AICAR	DMSO	tocris	150000
AM580	DMSO	tocris	2500
AS101	DMSO	tocris	12500
ATRA	DMSO	sigma	5000
BAI1	DMSO	tocris	5000
Bax inh pep V5	PBS	APEX Bio	30000
bpV (HOpic)	DMSO	selleckchem	5000
Calcitriol	DMSO	APEX Bio	10000
Capsaicin	DMSO	tocris	5000
CAY10566	DMSO	caymanchem	250
Celecoxib	DMSO	selleckchem	2500
CH223191	DMSO	selleckchem	2500
CHIR99021	DMSO	sigma	7500
Chroman1	DMSO	medchemexpress	250
Cintirorgon	DMSO	medchemexpress	5000
cyclosporin H	DMSO	sigma	5000
DAPT	DMSO	APEX Bio	5000
Dexamethasone	DMSO	tocris	2500
DMOG	DMSO	selleckchem	100000
Eltrombopag	DMSO	SCT	10000
Emricasan	DMSO	medchemexpress	5000
Eptifibatide	DMSO	sigma	5000
EPZ004777	DMSO	selleckchem	2500
Ferrostatin-1	DMSO	medchemexpress	2500
Forskolin	DMSO	tocris	5000
GDC-0449 (Vismodegib)	DMSO	APEX Bio	5000

bioRxiv preprint doi: https://doi.org/10.64898/2026.03.31.715684; this version posted April 1, 2026. The copyright holder for this preprint (which was not certified by peer review) is the author/funder, who has granted bioRxiv a license to display the preprint in perpetuity. It is made available under aCC-BY 4.0 International license.

GDC-0941	DMSO	selleckchem	1000
GNF351	DMSO	sigma	2500
Go 6983	DMSO	selleckchem	12500
GSK690693	DMSO	selleckchem	500
GW0742	DMSO	tocris	425
GW4869	DMSO	Sigma	10000
HX 531	DMSO	caymanchem	12500
IBMX	DMSO	selleckchem	50000
IDF-11774	DMSO	selleckchem	10000
iMAC2	DMSO	tocris	10000
ingenol	DMSO	selleckchem	25000
ionomycin	DMSO	tocris	2500
KF 38789	DMSO	tocris	15000
L-NAME	DMSO	tocris	25000
LDN193189	DMSO	sigma	6250
Leupeptin hemisulfate	DMSO	tocris	50000
LY294002	DMSO	selleckchem	12500
LY2955303	DMSO	tocris	10000
LY364947	DMSO	selleckchem	6250
MCC950	DMSO	selleckchem	500
Mifepristone	DMSO	tocris	10000
Nigericin	DMSO	selleckchem	250
NSC13128 (NAM)	DMSO	selleckchem	187500
NUTLIN-3A	DMSO	medchemexpress	10000
OAC1	DMSO	tocris	12500
PD169316	DMSO	selleckchem	5000
PD173074	DMSO	selleckchem	1000
PHA-L	PBS	invitrogen	500
Pifithrin-α	DMSO	tocris	10000
Pifithrin-μ	DMSO	tocris	10000
PKI 14-22 amide, myristoylated	DMSO	tocris	2500
PMA	DMSO	selleckchem	2500
PP1	DMSO	selleckchem	5000
PP242	DMSO	selleckchem	2500

bioRxiv preprint doi: https://doi.org/10.64898/2026.03.31.715684; this version posted April 1, 2026. The copyright holder for this preprint (which was not certified by peer review) is the author/funder, who has granted bioRxiv a license to display the preprint in perpetuity. It is made available under aCC-BY 4.0 International license.

PS48	DMSO	sigma	25000
PSB-12379	DMSO	medchemexpress	5000
Pyridone 6	DMSO	medchemexpress	500
(R)-(+)-Bay-K-8644	DMSO	medchemexpress	10000
rapamycin	DMSO	selleckchem	2500
refametinib	DMSO	selleckchem	2500
Ro5-3335	DMSO	tocris	10000
SAG	DMSO	selleckchem	250
SANT-1	DMSO	selleckchem	1000
SB203580	DMSO	tocris	10000
SB431542	DMSO	tocris	18000
SB590885	DMSO	selleckchem	2500
SC79	DMSO	selleckchem	10000
SR 0987	DMSO	selleckchem	12500
SR 7826	DMSO	tocris	2500
ST2825	DMSO	medchemexpress	20000
T0070907	DMSO	tocris	2500
tat-BECN1	DMSO	selleckchem	12500
trans-ISRIB	DMSO	tocris	1750
Tranylcypromine	DMSO	tocris	5000
TRULI (LATS-Inh1)	DMSO	selleckchem	10000
UM171	DMSO	selleckchem	400
UNC0224	DMSO	tocris	5000
Verteporfin	DMSO	selleckchem	12500
Y27632	DMSO	tocris	5000
YODA1	DMSO	selleckchem	2500
ZK159222	DMSO	caymanchem	1000
ZK164015	DMSO	tocris	1000
β-Estradiol	DMSO	sigma	1250
